# Synaptic Plasticity in Memristive Artificial Synapses and Their Robustness Against Noisy Inputs

**DOI:** 10.3389/fnins.2021.660894

**Published:** 2021-07-14

**Authors:** Nan Du, Xianyue Zhao, Ziang Chen, Bhaskar Choubey, Massimiliano Di Ventra, Ilona Skorupa, Danilo Bürger, Heidemarie Schmidt

**Affiliations:** ^1^Department Nano Device Technology, Fraunhofer Institute for Electronic Nano Systems, Chemnitz, Germany; ^2^Faculty of Electrical Engineering and Information Technology, Chemnitz University of Technology, Chemnitz, Germany; ^3^Department of Quantum Detection, Leibniz Institute of Photonic Technology, Jena, Germany; ^4^Institute for Solid State Physics, Friedrich Schiller University Jena, Jena, Germany; ^5^Analogue Circuits and Image Sensors, Universität Siegen, Siegen, Germany; ^6^Fraunhofer Institute of Microelectronics Circuits & Systems, ATTRACT Group Microelectronic Intelligence, Duisburg, Germany; ^7^Department of Physics, University of California, San Diego, La Jolla, CA, United States; ^8^Institute of Ion Beam Physics and Materials Research, Helmholtz-Zentrum Dresden-Rossendorf, Dresden, Germany

**Keywords:** artificial synapse, resistive switching, synaptic plasticity, neuronal noise, spike-timing dependent plasticity, cycle-number dependent plasticity, generalized frequency-dependent plasticity, unconventional neuromorphic computing

## Abstract

Emerging brain-inspired neuromorphic computing paradigms require devices that can emulate the complete functionality of biological synapses upon different neuronal activities in order to process big data flows in an efficient and cognitive manner while being robust against any noisy input. The memristive device has been proposed as a promising candidate for emulating artificial synapses due to their complex multilevel and dynamical plastic behaviors. In this work, we exploit ultrastable analog BiFeO_3_ (BFO)-based memristive devices for experimentally demonstrating that BFO artificial synapses support various long-term plastic functions, i.e., spike timing-dependent plasticity (STDP), cycle number-dependent plasticity (CNDP), and spiking rate-dependent plasticity (SRDP). The study on the impact of electrical stimuli in terms of pulse width and amplitude on STDP behaviors shows that their learning windows possess a wide range of timescale configurability, which can be a function of applied waveform. Moreover, beyond SRDP, the systematical and comparative study on generalized frequency-dependent plasticity (FDP) is carried out, which reveals for the first time that the ratio modulation between pulse width and pulse interval time within one spike cycle can result in both synaptic potentiation and depression effect within the same firing frequency. The impact of intrinsic neuronal noise on the STDP function of a single BFO artificial synapse can be neglected because thermal noise is two orders of magnitude smaller than the writing voltage and because the cycle-to-cycle variation of the current–voltage characteristics of a single BFO artificial synapses is small. However, extrinsic voltage fluctuations, e.g., in neural networks, cause a noisy input into the artificial synapses of the neural network. Here, the impact of extrinsic neuronal noise on the STDP function of a single BFO artificial synapse is analyzed in order to understand the robustness of plastic behavior in memristive artificial synapses against extrinsic noisy input.

## Introduction

The human brain can be considered as an advanced information storage and computation platform, capable of processing large volumes of real-time data in a massively parallel, fault-tolerant, and adaptive manner with extremely low energy consumption of ∼10 W (Townsley et al., 2020). Therefore, the biologically inspired neuromorphic computing paradigms are attracting significant interest as vehicles toward the implementation of real-time adaptive system for efficiently handling large amounts of data (Davies et al., 2018; Lin et al., 2020). The key to low-cost cognitive neuromorphic computing is the highly parallel processing offered by the large-scale synaptic connectivity between neurons (estimated ∼10^15^ synapses in a mammalian cortex) (Yang et al., 2018; Huang et al., 2021). The classical von Neumann architecture, however, has its memory bottleneck and is intrinsically different from the computational mode of the human brain from the computation architecture point of view (Neckar et al., 2018; Yang et al., 2019). Thus, in recent years, high-performance low-cost neuromorphic systems have been proposed employing unconventional non-von Neumann architecture inspired by the neural systems of the human brain (Pershin and Di Ventra, 2010; Akopyan et al., 2015; Thakur et al., 2018; Lin et al., 2020).

Neuromorphic computing based on non-Von Neumann architecture operates on the basis of hardware-neural-network (HW-NN) platforms consisting of numerous artificial synapses and neurons (Seo et al., 2020). The optimal candidate for mimicking synaptic activities is a device that can reproduce the complete functionality of biological synapses. The emerging nanoscale memristive devices are one of the most promising technologies enabling synaptic activities in neuromorphic systems (Jo et al., 2010; Huang et al., 2021). A memristive device (Nithya and Paramasivam, 2020) is a two-terminal element, whose resistance can be modulated between a low resistance state (LRS) and a high resistance state (HRS) (or among multiple resistance states) by applying appropriate external stimuli. The programmed resistance states are typically nonvolatile. The memristive devices also provide a number of other beneficial functional properties, including low power consumption, reconfigurability, fast switching speed, high endurance/retention, and excellent scalability (e.g., 3D integration manufacturing techniques) (Anusudha et al., 2020; Lin et al., 2020). For instance, memristive crossbar array with a 2-nm feature size and a single layer density up to 4.5 Tbit/in^2^ (Pi et al., 2019) has been demonstrated where the information density is comparable to 3D stacking in state-of-the-art 64-layer and multilevel 3D-NAND flash memory (Lee et al., 2018). Most recently, eight layers of monolithically integrated Ta/HafO_2_ memristive arrays were reported for a 3D convolutional neural network in applications of edge detection in video processing (Lin et al., 2020). These memristive devices attract wide attention and offer promising opportunities for emerging applications (Du et al., 2021) in highly efficient reconfigurable logic implementations (Tan et al., 2017; Xu N. et al., 2018; Luo et al., 2021), low-cost hardware security primitives (Mazady et al., 2015; Gao et al., 2018; Du et al., 2019) and chaotic oscillators (Li et al., 2018; Rajagopal et al., 2018; Singh et al., 2019). Especially, a memristive device intrinsically provides electrically tunable conductance, i.e., it enables updating of its conductance (artificial synaptic weight), upon electrical stimuli (neuronal activity), and demonstrates stable resistive states within its dynamic range (analog behavior) (Zhang et al., 2019; Huang et al., 2021). Such memristive artificial synapses show significant energy savings over traditional computing which involves separate processing of information and then storage into separate memory. A number of implementations of memristive artificial synapses based on different physical working mechanisms have been suggested which include inorganic redox switching devices (Abbas et al., 2018; Sokolov et al., 2020), metal ion migration switching devices (Yan et al., 2019; Zhang et al., 2019), phase change switching devices (Sarwat, 2017; Ren et al., 2018), ferroelectric switching devices (Kim and Lee, 2019; Li et al., 2020), and threshold switches (Wang et al., 2017; Kim and Lee, 2018; Sokolov et al., 2019). In most of these works, the neuromorphic devices are exploited to emulate one of the synaptic plastic behaviors, i.e., spike timing-dependent plasticity (STDP), cycle number-dependent plasticity (CNDP), spiking rate-dependent plasticity (SRDP), or long-term plasticity (LTP)/short-term plasticity (STP), and metaplasticity (Pedretti et al., 2017; Zang et al., 2017; John et al., 2018; Xu W.T. et al., 2018; Zhong et al., 2018; Guo et al., 2019; Kiani et al., 2019).

In this work, we comprehensively study the emulation of the long-term synaptic plasticities by using single BiFeO_3_ (BFO)-based memristive artificial synapses (Du et al., 2021), due to their unique functional properties, i.e., electroforming-free analog self-rectifying behavior. In a next step, several hundred BFO-based memristive artificial synapses and artificial neurons will be connected to form a NN platform. Typically, such NNs are prone to noise propagation. Therefore, we also study the robustness of plastic behavior in memristive artificial synapses against extrinsic noisy input. In the *Materials and methods* section, the ultrastable nonvolatile analog switching dynamic of BFO memristive artificial synapse is discussed. The waveforms with and without noisy input for studying the synaptic activities in this work are demonstrated. In the *Results* section, we present the experimentally recorded STDP, CNDP, and generalized frequency-dependent plasticity (FDP) in BFO memristive artificial synapse. We discuss their dependences on the memristive reconfigurability and neuronal activity in the applications in unconventional computing in the *Discussion* section. The demonstrated robustness against input noise as demonstrated for STDP will ensure high-level performance of the HW-NN platforms where BFO memristive devices are applied as artificial synapses.

## Materials and Methods

### Ultrastable Non-volatile Analog Resistive Switching

The BFO-based memristive devices are nonvolatile electroforming-free resistive switching devices (Du et al., 2018), which have drawn significant attention in the past decade due to their ultrastable multilevel analog switching properties (Du et al., 2013; Shuai et al., 2013) with long retention and highly stable endurance even at elevated temperatures (You et al., 2014). Previously, we have reported BFO-based memristive devices in emerging applications, such as reconfigurable logic (You et al., 2014) and hardware security primitives (Du et al., 2019). In this work, we utilize the BFO memristive devices for emulating the artificial synaptic activities upon the application of pre- and postsynaptic spikes based on various neuronal activities.

**FIGURE 1:**
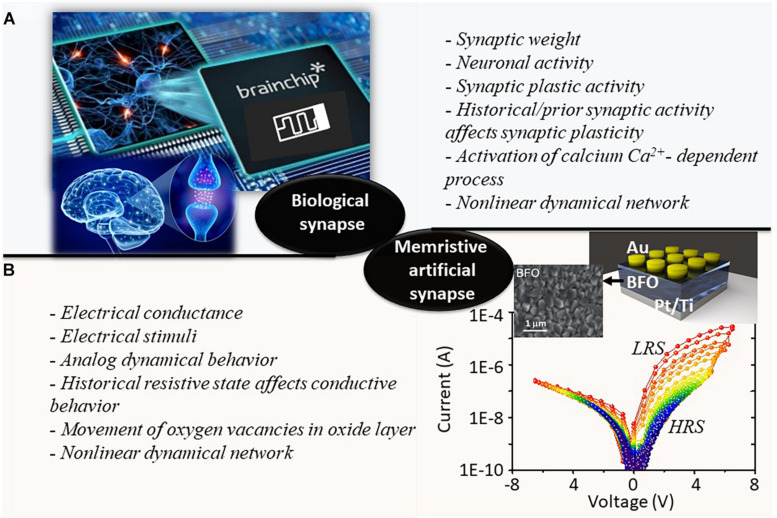
Comparative schematic illustrations of panels **(A)** biological synapse and **(B)** BFO-based memristive artificial synapse. The key terms representing the nonlinear dynamical processes during the information transmission in the biological and artificial synapses are summarized and listed in each corresponding subfigure. The current–voltage (*I*–*V*) characteristics of BFO memristive devices under multiple linear sweeping biases of −*V*_*max*_ → +*V*_*max*_ →−*V*_*max*_ are demonstrated in panel **(B)** with *V*_*max*_ = 2, 2.3, 2.6….6.2, 6.5 V. The colors of *I*–*V* curves are changing from blue color to red color with increasing biases. The topographic SEM image of the surface of BFO thin film is illustrated beside the schematics of BFO-based memristive device.

As illustrated in Figure 1A of the biological human brain, the various synaptic plastic activities are governed by the different neuronal activities in response to changing environments, where the synaptic weights are defined not only by the neuronal action functions but also by the historical synaptic activities. Thus, the nonlinear dynamical network is established. Figure 1B demonstrates schematics of BFO-based artificial synapses. The polycrystalline BFO thin films are fabricated by pulsed laser deposition on Pt/Ti/SiO_2_/Si substrates (Shuai et al., 2013; Du et al., 2018). The nominal thickness of BFO thin film is 500 nm. The circular Au top contacts with a thickness of 180 nm are magnetron sputtered on the BFO thin film. The *I*–*V* characteristics of the proposed BFO-based artificial synapse are recorded by applying the sweeping source voltage from −6.5 V → +6.5 V →−6.5 V between the Au top electrode and the bottom electrode. Moreover, multiple cycles of linear sweeping with the maximum amplitude *V*_*max*_ = 2, 2.3, 2.6…. 6.2 V are also plotted in Figure 1B. The *I*–*V* characteristics were recorded using a Keithley SourceMeter 2400. The duration of each bias value amounts to 100 ms. The physical mechanism underlying analog resistive switching dynamics observed in BFO memristive devices is related to the nonvolatile change of flexible barriers in the Ti-containing BFO/Pt/Ti interface region (bottom electrode region, BE region), whereas a Schottky diode with a fixed barrier height is formed at the Au/BFO interface region (top electrode region, TE region). By applying positive writing bias (SET process) to TE of the memristive device, the mobile oxygen vacancies are attracted to the BE region and effectively trapped by Ti donors, which can lower the barrier height at the interface between the BFO layer and BE. With the nonrectifying BE region and rectifying TE region, the memristive device exhibits rectifying behavior in LRS. By applying negative writing bias (RESET process) to the TE of the memristive device, the mobile donors can be homogeneously distributed within the BFO thin film, with both TE and BE regions demonstrating rectifying behavior, and hence, the device is in HRS.

The nonlinear switching dynamic in BFO memristive device shows a number of characteristics that make it well suited for applications as an artificial synapse in brain-inspired neuromorphic computing systems (i.e., in HW-NN platform). For instance, (1) the electrical conductance in nonvolatile BFO memristive device is defined not only by the electrical stimuli that are applied to the TE and BE of device but also by its historical resistive state. (2) The complex ultrastable multilevel switching behavior as demonstrated in Figure 1B from BFO memristive device ensures that up to 8-bit analog resolution can be reliably programmed in the device (Shuai et al., 2013). (3) The exponential relationship converged between the stepping DC voltage and electrical conductance (Mayr et al., 2012) makes it conform closely to the ideal spike timing-dependent plastic behavior observed from biological synapses (Bi and Poo, 1998). (4) Most of the memristive devices require one electroforming step (Yang et al., 2009) upon the manufacturing process, where a stronger electrical field (much stronger than in the device’s regular operation) initiates the formation of a conductive filament, bringing the device into the low-resistance state. By contrast, the electroforming-free BFO-based memristive devices require no electroforming process, which are desired in general due to their potential high yield and long-term reliability of memristive cells. (5) By leveraging the electroforming-free and self-rectifying behaviors, the BFO memristive device can be employed for constructing reliable selector-free crossbar arrays in the HW-NN system. The high-ohmic region defines a readout region where only one single cell can be actively addressed in crossbar array. This effectively eliminates the multiple sneak path current issues (Jung et al., 2021).

### Synaptic and Neuronal Activities

Synaptic plasticity is a process for modifying the connection strength between the pre- and postsynaptic neurons in response to generated paired neuronal impulse. STDP and SRDP are both fundamental Hebbian synaptic activities discovered in mammalian hippocampus and the neocortex (Hebb, 1949), which demonstrate most prominent learning and memory behaviors in the brain cognitive system. In STDP, the well-defined timing of pre- and postsynaptic spikes determines the direction and strength of synaptic plasticity, whereas in SRDP, the presynaptic firing rate defines the sign and magnitude of synaptic plasticity.

**FIGURE 2:**
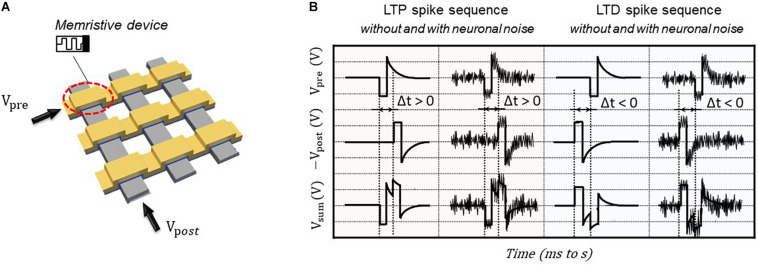
Illustrative schemes for memristive artificial synapse and STDP driving voltage waveforms. **(A)** Circuit demonstration for STDP implementation by applying pre- and postsynaptic voltages to TEs and BEs to one memristive artificial synapse in crossbar array, respectively. **(B)** Quasi-static stimulation protocol applied to TE of BFO memristive artificial synapse for emulating LTP and LTD learning rules without and with neuronal noises.

The emerging memristive crossbar array (Figure 2A) can provide a promising hardware realization of brain-inspired neuromorphic computing system due to their intrinsic functional properties, i.e., parallel processing capability, excellent scalability, and low power consumption. In the memristive crossbar array, the memristive devices at each cross-point are used to emulate the artificial synapse. The quasi-static stimulation protocol (Du et al., 2015) is applied for generating the single paring STDP learning functions on BFO-based artificial synapse, which consists of three steps—memory initialization, single pairing spike sequence, and memory consolidation. By applying well-defined single pairing spike sequence, including presynaptic waveform *V*_pre_ and postsynaptic waveform *V*_post_ as demonstrated in Figure 2B to the TE and BE of BFO memristive artificial synapse, the associative synaptic plasticity learning rule STDP can be emulated and recorded. Each pre- and postsynaptic waveform consists of one rectangular pulse (with pulse width of *t*_p_ and pulse amplitude of *V*_p_) and one exponentially decaying pulse *V*_exp_:

(1) $${\text{V}}_{e\,x\,p}=\left|{\text{V}}_{p}\right|\cdot {\text{e}}^{-t/\tau },$$

with the decay time τ = τ_pre_ = τ_post_ =  2.5⋅*t*_p_, where τ_pre_ and τ_post_ are the exponential decay times of pre- and postsynaptic waveforms.

As demonstrated in Figure 2B, the positive delay time Δ*t* between the pre- and postsynaptic neurons leads to long-term potentiation (LTP), which exhibits the long-term enhancement of synaptic excitatory strengths, whereas the negative delay time Δ*t* between the pre- and postsynaptic neurons results in long-term depression (LTD), which is reversal of LTP, and reveals the long-term weakening of them. The memory initialization and memory consolidation are applied prior to and after single paring LTP/LTD spike sequences in Figure 2B, respectively. During memory initialization process, the negative writing pulse *V*_w_ = −6 V is applied prior to LTP spike sequences for resetting memristive cells into HRS (RESET process), while the positive writing pulse *V*_w_ = +6 V is used prior to LTD spike sequence for setting memristive cells into LRS (SET process). Such RESET and SET processes are important for memristive applications in general for defining the initial states of memristive cells as well as for further recording the comparable experimental results. Finally, memory consolidation can be investigated for accessing the feature property of long-term stabilization in synaptic weight by introducing different waiting times before recording the synaptic weight. In our previous work, the single pairing STDP learning functions under synaptic spike sequences with varying pulse widths and varying waiting time are studied (Du et al., 2015). In this work, the spike sequences with different pulse widths *t*_p_ or with different pulse amplitudes *V*_p_ are selected for implementing single pairing STDP learning rules on BFO-based artificial synapse. In this work, the waiting time of 2 s for memory consolidation is kept unchanged. Due to the analog switching behavior of BFO memristive devices, there is no abrupt change of current during switching of the device between HRS and LRS. According to the empirical electrical testing of memristive cells, there is no current change occurring while continuously applying the bias 2 V on BFO cells. Thus, the rectangular pulse of 2 V is defined as the reading bias of BFO memristive artificial synapse for recording resistance values at various memristance states. In addition, the LTP and LTP spike sequences with pulse amplitudes 3.75, 3, and 2 V are chosen and implemented for recording the STDP learning functions. As demonstrated in Figure 2B, *V*_sum_ = *V*_pre_ - *V*_post_, we expect more significant changes in LTP current and LTD current when applying spike sequences with pulse amplitudes of 3.75 and 3 V than that of 2 V. This is so as the amplitude of superimposed spike sequence *V*_sum_ is much higher than the normal reading bias of the device. One may note that the choice of pulse amplitude should also not be too high to break down the device. In this case, the breakdown bias of used BFO memristive device is around ± 10 V. Particularly, in this work, the STDP learning functions under spike sequences without and with noisy input up to 30% of selected pulse amplitude are comparably investigated.

Furthermore, the presynaptic spike trains under different firing rates are applied to the BFO memristive artificial synapses for emulating SRDP (Rachmuth et al., 2011), which is also one of the most important synaptic learning mechanisms in brain cognitive behaviors. In comparison with the traditional stimulation protocol for emulating SRDP learning rules (where only the spiking rates of spike trains are varying), we have analyzed the synaptic weight change and excitatory postsynaptic current (EPSC) in dependence on the proportional relationship between *t*_p_ and *t*_int_ within the same frequency range, which is termed as generalized FDP in this work. Here, the *t*_p_ and *t*_int_ represent the pulse width and interval time in one pulse cycle of presynaptic spike trains, respectively. The FDP study utilizes the application of well-defined spike trains with *t*_p_ = *t*_int_ scheme, varying *t*_p_ scheme, and varying *t*_int_ scheme to BFO memristive artificial synapse. The generalized FDP study is helpful for in-depth understanding of the impact of learning and memory modulations on BFO memristive artificial synapse.

Besides STDP and FDP, the synaptic plasticity induced by the accumulation of cycle number of prespikes, i.e., CNDP, is also emulated in BFO memristive artificial synapse. CNDP is recorded by applying the consecutive presynaptic spikes with different spike numbers to the TEs of BFO memristive artificial synapses, which is considered as the most basic test for enabling both the training and testing processes in the HW-NN system. The aforementioned synaptic plastic behaviors, i.e., STDP, FDP, and CNDP, differ in their learning capabilities. However, all of the introduced electrical stimulation protocols activate the permanent long-term learning behaviors in BFO memristive artificial synapse in this work. The temporary short-term synaptic plasticity is not considered here. Note that the initialization step is required upon each synaptic plastic test, which refers to the application of a writing pulse *V*_w_ = |6 V| to set the BFO device into predefined determinative high or low resistive states. Each synaptic weight demonstrated in FDP and CNDP learning diagrams is an average value of five conductive values during each spike.

## Results

### Spike Timing-Dependent Plasticity in Dependence of V_p_ and t_p_

**FIGURE 3:**
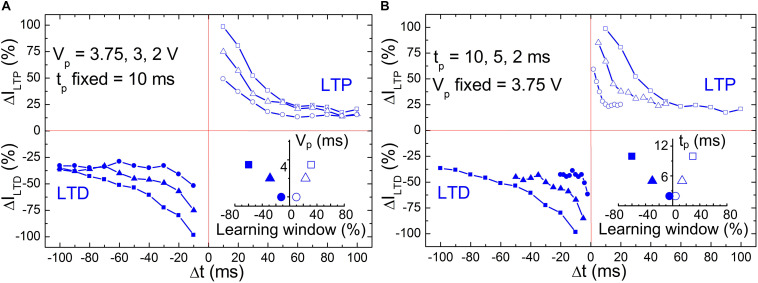
Comparison of STDP diagrams **(A)** with different pulse amplitudes *V*_p_ = 3.75 V (square), 3 V (triangle), and 2 V (circle) and same pulse width *t*_p_ = 10 ms (i.e., learning window of *τ* = 25 ms) and **(B)** with different pulse width *t*_p_ = 10 ms (square), 5 ms (triangle), 2 ms (circle) and the same pulse amplitude *V*_p_ = 3.75 V. The measurement waiting time is defined as *t*_w_ = 2 s. The inset of panel **(A)** shows the length of learning windows in dependence of the pulse amplitude *V*_p_, and the inset of panel **(B)** shows the length of learning windows in dependence of pulse width *t*_p_.

In a previous work, we have demonstrated that STDP can be emulated on BFO artificial synapses by applying 60–80 pairings (Mayr et al., 2012; Cederström et al., 2013) or single pairing of pre- and post-synaptic spikes with a significant wide range of timescale configurability (Du et al., 2015). Figure 3 shows the STDP diagrams in BFO artificial synapse exclusively in dependence on two input parameters: pulse amplitude (Figure 3A) and pulse width (Figure 3B). During the single pairing STDP measurement shown in Figure 3A, we kept the pulse width *t*_p_ as 10 ms with a learning window of *τ* = 25 ms. We varied the pulse amplitude by 3.75, 3, and 2 V. After applying potentiating and depressing spike sequence, both LTP current *I*_*LTP*_ and LTD current *I*_*LTD*_ are recorded under reading bias at 2 V. The initialization bias with writing amplitude *V*_w_ = |6 V| has been chosen to RESET and SET the BFO memristive artificial synapse prior to the potentiating and depressing spike sequences. The normalized LTP current Δ*I*_*LTP*_ and LTD current Δ*I*_*LTD*_ are then plotted against the spike timing differences from |Δ*t*| = *t*_p_ up to |Δ*t*| = 10^∗^*t*_p_. The decreased insufficient spike amplitudes (i.e., *V*_p_ = 3 V, 2 V) result in the reduction of normalized current in both potentiation and depression regions in comparison with spike amplitude of *V*_p_ = 3.75 V. At |Δ*t*| = *t*_p_, the Δ*I*_*LTP*_/Δ*I*_*LTD*_ is dramatically depressed at decreased spike amplitudes of *V*_p_ = 3 and 2 V. The saturated Δ*I*_*LTP*_/Δ*I*_*LTD*_ is evaluated by comparing the mean value of Δ*I*_*LTP*_/Δ*I*_*LTD*_ in the saturation region, which is defined from |Δ*t*_*s*_| up to 100 ms/−100 ms. Under spike amplitudes of *V*_p_ = 3.75 V, the saturated Δ*I*_*LTP*_/Δ*I*_*LTD*_ amounts to 19.8% (Δ*t*_*s*_ = 70 ms)/−36.2% (Δ*t*_*s*_ = −100 ms). In comparison to that, under decreased spike amplitudes of *V*_p_ = 3 and 2 V, STDP learning functions saturate faster at slightly decreased saturated Δ*I*_*LTP*_/Δ*I*_*LTD*_ as illustrated on the left side of Table 1. Herein, the saturated Δ*I*_*LTP*_/Δ*I*_*LTD*_ amounts to 18.4% (Δ*t*_*s*_ = 60 ms)/−35.5% (Δ*t*_*s*_ = −70 ms) and 14.3% (Δ*t*_*s*_ = 50 ms)/−33.2% (Δ*t*_*s*_ = −30 ms), respectively.

**TABLE 1:** The saturated Δ*I*_*LTP*_/Δ*I*_*LTD*_ in the saturation region (from | Δ*t*_*s*_| up to 10 * *t*_p_) recorded upon the application of potentiation/depression spike sequence with different pulse amplitudes of *V*_p_ = 3.75, 3.00, and 2.00 V (*t*_p_ is kept constant as 10 ms) or with *t*_p_ = 10, 5, and 2 ms (*V*_p_ is kept constant as *V*_p_ = 3.75 V).

**V_p_ (V)**	**Δ*I*_*LTP*_ (%) at Δ*t*_*s*_**	**Δ*I*_*LTD*_ (%) at Δ*t*_*s*_**	**t_p_ (ms)**	**Δ*I*_*LTP*_ (%) at Δ*t*_*s*_**	**Δ*I*_*LTD*_ (%) at Δ*t*_*s*_**
3.75	19.8 at 70 ms	−36.2 at −100 ms	10	21.3 at 70 ms	−41.1 at −70 ms
3.0	18.4 at 60 ms	−35.5 at −70 ms	5	21.1 at 70 ms	−45.5 at −70 ms
2.0	14.3 at 50 ms	−33.2 at −30 ms	2	24.5 at 70 ms	−43.8 at −70 ms

Contrary to Figure 3A, we kept pulse amplitude unchanged in Figure 3B and have chosen different pulse widths *t*_p_ as 10, 5, and 2 ms. Due to the shortened pulse widths with 5 and 2 ms, the learning time constant of STDP function *τ* (*τ* = 2.5 ^∗^ *t*_p_) is adjusted as 12.5 and 5 ms, and the overall spike timing range for both LTP and LTD branches is confined within 50 and 20 ms, respectively. At |Δ*t*| = *t*_p_, the normalized LTP current Δ*I*_*LTP*_ and LTD current Δ*I*_*LTP*_ are significantly decreased due to the decreased *t*_p_. However, the saturation of Δ*I*_*LTP*_/Δ*I*_*LTD*_ is starting from |Δ*t*_*s*_| = 7 ^∗^ *t*_p_ up to 10 ^∗^*t*_p_, and the saturated value is comparable among the chosen spike width *t*_p_ = 10, 5, and 2 ms as illustrated on the right side of Table 1, i.e., Δ*I*_*LTP*_ = 21.3, 21.1, and 24.5% in the potentiation region, and Δ*I*_*LTD*_ = −41.1, −45.5, and −43.76% in the depression region, respectively.

The learning windows at Δ*I*_*LTP*_/|Δ*I*_*LTD*_| = 50% are shown in the insets of Figures 3A,B in dependence of pulse amplitude *V*_p_ and pulse width *t*_p_, respectively. In both cases, the increase of learning windows at Δ*I*_*LTP*_/|Δ*I*_*LTD*_| = 50% with respect to *V*_p_ and *t*_p_ can be observed, where the increase velocity in the LTP region is larger than that in the LTD region.

### Cycle Number Dependent Plasticity in Dependence of V_p_

**FIGURE 4:**
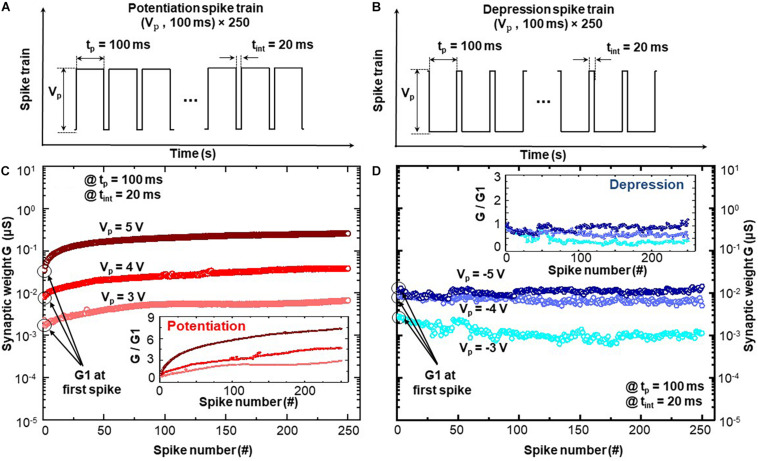
Cycle number-dependent plasticity in BFO artificial synapse: **(A)** potentiation and **(B)** depression spike sequence as electrical stimuli applied to BFO artificial synapse with pulse width t_*p*_ = 100 ms and various pulse amplitudes of V_*p*_. The time interval between two pulses is defined as t_*int*_ = 20 ms. Synaptic weights of panels **(C)** potentiation and **(D)** depression dynamics under 250 identical potentiation and depression spikes with pulse amplitudes of V_*p*_ = 3, 4, and 5 V. Insets in panels **(C,D)** demonstrate the synaptic change of potentiation and depression dynamics: Synaptic weight G normalized with the synaptic weight G1 recorded at first spike.

Cycle number dependent plasticity suggests that the consecutive stimuli enable the incremental modification of synaptic weight (electrical conductance) in BFO-based artificial synapse. Figure 4 demonstrates the examination of CNDP functionality upon application of an initialization step: the potentiation spike train shown in Figure 4A requires an initialization pulse for the RESET process with amplitude of −6 V, while the depression spike train of Figure 4B requires one for the SET process with an amplitude of 6 V. After the initialization step, the corresponding potentiation and depression spike trains have been applied to the BFO memristive artificial synapse in analogy to the process wherein the presynaptic spikes stimulate the synapse. During the CNDP test, the spike amplitude is set as *V*_p_ ≥ 3 V to ensure the synaptic weights in BFO memristive artificial synapse can be permanently changed (long-term learning rules) under the spike sequence with spike width of 100 ms (with time interval 20 ms).

Figures 4C,D demonstrate the CNDP synaptic weights (i.e., the memristive conductance) after applying a potentiation and depression spike sequence consisting of 250 spikes to the top electrode of BFO memristive artificial synapse, respectively. During the application of one spike stimulus, five conductance values are recorded over the memristance device. One CNDP synaptic weight is computed as an average value of five conductance values recorded during each spike. By applying potentiation spike sequence, Figure 4C indicates that the synaptic weights of BFO artificial synapse increase gradually with the applied spike number, i.e., long-term potentiation. On the other hand, Figure 4D reveals gradually decreased synaptic weights under depression spike sequence, i.e., long-term depression. The insets in Figures 4C,D demonstrate the normalized synaptic weight change, where the overall synaptic weight is divided by the first conductance value G1 recorded at first spike. Under potentiation spike sequence, the higher spike amplitude with *V*_p_ = 5 V leads to a more significant increase in synapse weight in comparison with *V*_p_ = 3 V and *V*_p_ = 4 V. It is so because more oxygen vacancies are driven into the BE direction and lower the barrier height at the BFO/Pt interface during the application of spike train with *V*_p_ = +5 V. Under the depression spike sequence, the no obvious change can be found under the spike train with spike amplitude *V*_p_ = −5 V, as the amplitude of −5 V causes the simultaneous switching of memristive device into HRS (no switching of intermediate state possible). The significant change of synapse weight can only be induced by the spike sequence with a lower amplitude, i.e., *V*_p_ = −3 V or *V*_p_ = −4 V. Such observation indicates that the dynamical range of BFO memristive device under negative bias range is smaller than that under positive bias range. The negative pulses with the same pulse amplitude lead to a faster switching into HRS than positive pulses into LRS, and it means that the HRS is a preferable state in BFO memristive state. Therefore, one can conclude from Figure 4 that synaptic weight of BFO artificial synapse can be continuously adjusted by presynaptic spikes and is highly dependent on the spike amplitude and cycle number of spikes, which is suitable for application in HW-NN.

### Frequency-Dependent Plasticity in Dependence of t_p_ and t_i*nt*_

The generalized FDP studied here describes a feature of memristive artificial synapse that the synaptic weight (conductivity) not only changes with the applied presynaptic firing rate but is also strongly related to the variation of pulse width *t*_p_ and time interval *t*_int_ within each spike cycle of presynaptic spike trains, i.e., *t*_p_ = *t*_int_ scheme, varying *t*_p_ scheme and varying *t*_int_ scheme. An initialization pulse for the RESET process with an amplitude of −6 V is applied to BFO memristive artificial synapse prior to each spike train.

**FIGURE 5:**
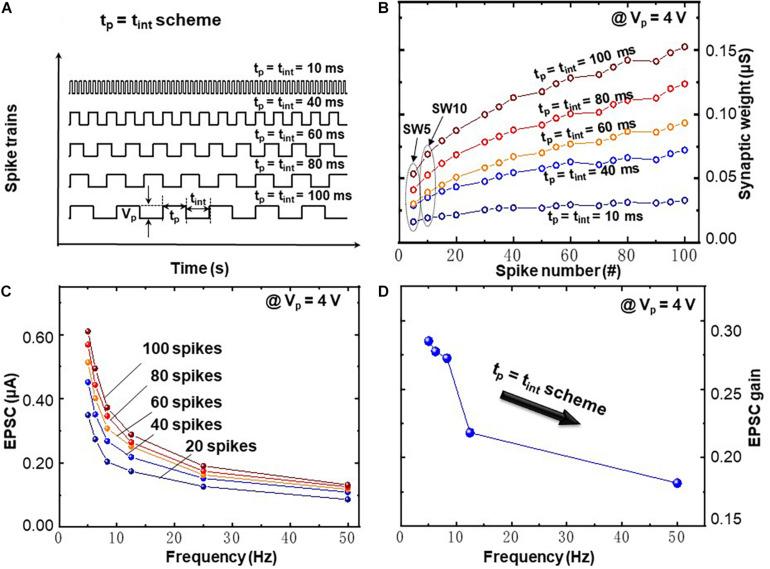
Frequency-dependent plasticity learning rules executed by spike trains with synchronous variation of pulse width t_p_ and time interval t_*int*_ (t_*p*_ = t_*int*_ scheme). **(A)** Schematic diagram of spike trains with amplitude of V_p_ = 4V and synchronous variation of t_p_ = t_*int*_ = 10, 40, 60, 80, and 100 ms. **(B)** Recorded synaptic weights as a function of spike number upon 100 consecutive spikes. **(C)** EPSC response triggered by spike trains (V_p_ = 4V) and different frequencies. **(D)** Frequency-dependent EPSC gains at V_p_ = 4V.

Figure 5 demonstrates the FDP with *t*_p_ = *t*_int_ scheme, where the frequency dependence in presynaptic spike train is caused by a synchronous variation of time interval *t*_int_ and pulse width *t*_p_ (*t*_int_ = *t*_p_ = 10, 40, 60, 80, and 100 ms, as illustrated in Figure 5A). Thus, the studied frequency range is 5.0, 6.3, 8.3, 12.5, and 50.0 Hz, respectively. Each spike train contains 100 pulses with spike amplitude of 4 V and applied to the top electrode of memristive artificial synapse. A distinct feature can be recognized in Figure 5B as the gradual increment of the average synaptic weights along the increasing spike number. The same feature is observed in the CNDP learning rules in Figure 4C, i.e., the synaptic weight is gradually increased under potentiation spike trains. Besides that, a clear dependence between *t*_p_ = *t*_int_ and synaptic weight is visible, namely, the larger *t*_p_ = *t*_*i*nt_ leads to a more significant conductance enhancement. Figure 5C shows the frequency-dependent EPSC response where a strong decrement of EPSC values with increased frequency is visualized. Under spike trains with higher frequency (with the same spike number), less oxygen vacancies can be activated and driven to the BE region which results in less EPSC response. Figure 5D demonstrates frequency-dependent EPSC gain. The EPSC gain is computed as (SW10 − SW5)/SW5, where SW5 and SW10 represent the 5th (SW5) and 10th (SW10) EPSC values as illustrated in Figure 5B, respectively. The EPSC gain decreases from 0.29 to 0.18, while the frequency increases from 5.0 to 50.0 Hz. This indicates that the BFO memristive artificial synapse exhibits decremental frequency-dependent synaptic response characteristics in FDP implementation with *t*_p_ = *t*_int_ scheme.

**FIGURE 6:**
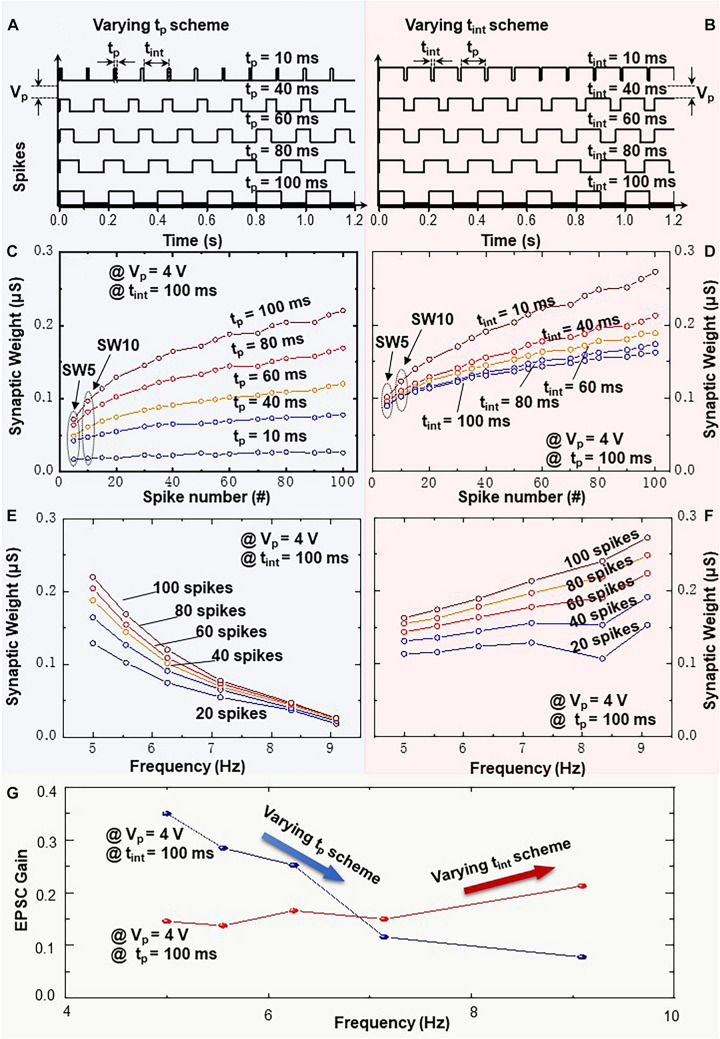
Frequency-dependent plasticity learning rule executed by spike trains with variation of pulse width t_p_ or time interval t_*int*_ = 10, 40, 60, 80, and 100 ms (varying t_p_ scheme and varying t_*int*_ scheme). Schematic diagrams of spike trains with amplitude of V_p_ = 4V and variation of panels **(A)** t_p_ or **(B)** t_*int*_. Recorded synaptic weights as a function of spike number upon 100 consecutive spikes with **(C)** t_p_ or **(D)** t_*int*_ variation. EPSC response triggered by spike trains (V_p_ = 4V) and different frequencies with **(E)** t_p_ or **(F)** t_*int*_ variation. **(G)** Frequency-dependent EPSC gains at V_p_ = 4V with t_p_ or t_*int*_ variation.

In Figure 6, a single variation of pulse width *t*_p_ or time interval *t*_int_ is induced in the presynaptic spike trains, i.e., varying *t*_p_ scheme or varying *t*_int_ scheme. These are applied to the top electrode of memristive artificial synapse. In order to study the individual impact of *t*_p_ and *t*_int_ on synaptic weight change, we set one of the two variables in the spike train (*t*_int_ or *t*_p_) varying from 10 to 100 ms and fixed the other one unchanged. In both cases, the examined frequencies of the presynaptic spike trains are the same at 5.0, 5.6, 6.3, 7.2, and 9.1 Hz. Each spike train contains 100 pulses with spike amplitude of 4 V. In the varying *t*_p_ scheme or in the varying *t*_int_ scheme, the synaptic weight increases along with the increasing spike numbers as demonstrated in Figures 6C,D. More significant increments in synaptic weight can be recorded at larger pulse width *t*_p_ in the varying *t*_p_ scheme or at smaller time interval *t*_int_ in the varying *t*_int_ scheme, respectively. Highlighted in Figure 6C, the initial synaptic weights under spike trains with varying *t*_p_ are distributed in a discrete state. On the other hand, the recorded synaptic weights in Figure 6D are gathered together at the initial points. It is noteworthy that in both cases, the average synaptic weights under spike trains with varying variables (*t*_p_ or *t*_int_) are sequentially recorded from 10 up to 100 ms upon one single initialization step with amplitude of −6 V. It is revealed that the varying *t*_p_ scheme leads to a higher current level at *t*_p_ = 100 ms and *t*_int_ = 100 ms in comparison with the varying *t*_int_ scheme. Such observation can be attributed to the accumulated impact of historical current flow on the actual conductance level of BFO memristive device. Figures 6E,F demonstrate the gradual decrement and increment of EPSC response within the same frequency range, which indicates that the synaptic weights of BFO memristive artificial synapse can be weakened or enhanced within the same frequency range, while the frequency variation is only caused by changing the proportional relationship between *t*_p_ and *t*_int_ in one spike cycle.

Figure 6G demonstrates the frequency-dependent EPSC gain. The EPSC gain is computed as (SW10 − SW5)/SW5, where SW5 and SW10 represent the 5th (SW5) and 10th (SW10) average EPSC values as illustrated in Figures 6C,D, respectively. The EPSC gains in Figure 6G indicate that the BFO memristive artificial synapse can exhibit both incremental and decremental frequency-dependent synaptic response characteristics in FDP implementation with *t*_int_ varying and *t*_p_ varying schemes, respectively. It is noteworthy that the stimulation protocol for FDP with varying *t*_int_ scheme corresponds to SRDP in biological synapses. It suggests that the synaptic weight in biological synapse is highly dependent on the presynaptic spiking rate, and hence, more frequent stimulation leads to a large change of synaptic weight (Mori et al., 2004; Froemke et al., 2006). In biological systems, the duration of a single spike is considered invariable. Only the time interval between spikes influences the spiking rate, thus leading to the modification of synaptic weight. However, in the experimental study of BFO memristive artificial synapse, the modification of artificial synaptic weight can be modified not only by time interval *t*_int_ between spikes but also by the spike pulse width *t*_p_, which is essential for understanding the competition between the synaptic excitation and memory consolidation processes in the long-term learning rules.

### Impact of Noisy Input on STDP

**FIGURE 7:**
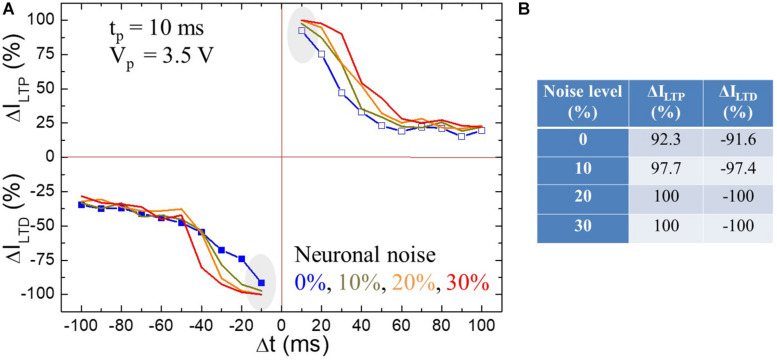
**(A)** Impact of triangle neuronal noise with different noise amplitudes *V*_nn_, i.e., *V*_nn_ = 0 V (blue line), 0.35 V (dark yellow line), 0.70 V (orange line), and 1.05 V (red line) at *f*_nn_ = 593 Hz on STDP learning functions in BFO artificial synapse. The STDP diagram has been recorded with pulse width *t*_p_ = 10 ms (i.e., learning window of *τ* = 25 ms) and pulse amplitude *V*_p_ = 3.50 V. The measurement waiting time is defined as *t*_*w*_ = 2 s. **(B)** Illustration of the experimental recorded normalized LTP/LTD current Δ*I*_*LTP*_/Δ*I*_*LTD*_ at | Δ*t*| = *t*_p_ = 10 ms (gray marked) at neuronal noise level of 0, 10, 20, and 30%.

We now demonstrate the robustness of STDP against extrinsic noisy input. Chen et al. (2014b) studied the noise of micropipette amplifiers for extracellular neural recordings from dead and live animals. Data from neural recordings may be fed into the HW-NN being part of neuroimplants. The artificial synapses of the HW-NN should be robust against noise. Chen et al. found that the two dominant noise sources degrading the neural voltage signal in the recordings is the intrinsic noise of the amplifier and the thermal noise of the glass pipette. The measured overall noise level in dead and live animals was 6 and 35%, respectively. Figure 7A shows LTP and LTD learning functions in BFO artificial synapse and demonstrates the robustness of STDP of BFO memristive artificial synapses up to a noise level of 30%. In order to provide the first rough estimation for the impact of noisy input on STDP learning behavior in memristive artificial synapses, the additive neuronal noise in this work has been estimated by the triangular pulse under frequency *f*_nn_ = 593 Hz. Such triangular neuronal noise with noise level up to 30% is attached on the potentiating and depressing spike sequences and applied to BFO memristive artificial synapse. As the spike sequences, we have chosen pulse width *t*_p_ as 10 ms (learning window of *τ* = 25 ms) and decreased pulse amplitude as *V*_p_ = 3.5 V. This ensures that the exponential-like decay of the normalized current is dominated for both LTP and LTD learning functions, while the superimposed neuronal noise of pre- and postspikes with noise levels from 10 up to 30% would not cause breakdown of the memristive device. The initialization bias *V*_w_ = |6 V| has been chosen to RESET and SET the BFO memristive artificial synapse, and both LTP current *I*_*LTP*_ and LTD current *I*_*LTD*_ are recorded under reading bias 2 V.

Upon the pre- and post-synaptic spikes associated with neuronal noise, we retain a graded weight as demonstrated in Figure 7A. Due to the insufficient spike bias *V*_p_ = 3.5 V without neuronal noise, the normalized LTP/LTD current at |Δ*t*| = *t*_p_ = 10 ms amounts to 92.3%/−91.6%, whereas the normalized LTP/LTD current amounts to 97.7%/−97.4%, 100%/−100%, and 100%/−100% under LTP/LTD spike sequences with noise levels of 10, 20, and 30%, respectively (as listed in the table in Figure 7B). Thus, the normalized STDP current saturates at spike timing of |Δ*t*| = *t*_p_ = 10 ms, which indicates that the memristive device has been fully switched to LRS/HRS under enhanced LTP/LTD spike sequences due to the additive noise amplitudes of 0.7 V (noise level of 20%) and 1.05 V (noise level of 30%) despite the insufficient original pulse amplitude of 3.5 V. In the spike timing range of 0 < *t*_p_ < |Δ*t*| ≤ 10 ^∗^ *t*_p_, an exponential decrease dominates against the STDP learning curves with increasing delay time |Δ*t*| and finally stabilizes at Δ*I* values, where no noise is applied.

## Discussion

Biological intelligence is based on learning and memorization. Learning and memory are emergent synaptic plastic behaviors governed by modifications in neuronal activities in response to changing environments. The STDP, CNDP, and FDP (including SRDP) belong to the classic synaptic learning mechanisms in brain cognitive behaviors.

### Synaptic Plasticity Induced by Memristive Reconfigurability

In this work, the waveform-defined single pairing STDP in BFO-based artificial synapses has been demonstrated, where the direction and strength of synaptic plasticity are determined by the well-defined timing of pre- and postsynaptic spikes. Prior literature has demonstrated that STDP learning functions as device-inherent behavior (Ohno et al., 2011) with fairly small learning window or considerable high statistical variations (Jo et al., 2010; Alibart et al., 2012). In comparison to that, with the help of ultrastable analog switching behavior of BFO memristive devices, this work enables the deterministic weight change under signal pairing pre- and postspikes and fulfills highly configurable, finely grained learning curves. As demonstrated in Figure 3, by exploiting memristive massive dynamical tunability, not only the timescale configurability but also the amplitude configurability of STDP learning window is fulfilled due to their multilevel programming capability.

In biological synaptic study, the definitions of short-term plasticity and long-term plasticity are made based on observations that the modification of synaptic weight in synapse can be either temporary or permanent (Saïghi et al., 2015). In CNDP implementation in Figure 4, the pulse amplitudes of 3, 4, and 5 V are applied, to ensure that only long-term plasticity is activated in BFO memristive artificial synapses. Thus, the memristive reconfigurability revealed in CNDP suggests that the synaptic weights in BFO memristive artificial synapse can be gradually incremented or decremented using consecutive positive or reverse biased spikes and stored according to the long-term learning rules. Further beyond SRDP, the systematical and comparative study on generalized FDP is carried out, which reveals that synaptic activity not only depends on firing rate but also depends on the proportional relationship between *t*_p_ and *t*_int_. In FDP implementation, the pulse amplitude of 4 V is chosen for emulating the long-term synaptic plasticity under the *t*_p_ = *t*_int_ scheme, varying *t*_p_ scheme, and varying *t*_int_ scheme as demonstrated in Figures 5, 6. According to the CNDP learning features emulated in Figure 4, the comparable FDP learning tendency under the pulse amplitudes of 3 and 5 V can also be expected.

### Synaptic Plasticity in Dependence of Neuronal Activity

Synaptic plasticity is a form of biological learning process (e.g., Hebbian learning), and the excitability of individual synaptic cell is affected by the interplay of both synaptic intrinsic and neuronal network modulations.

The generalized FDP implementation in Figure 6 reveals that within the same presynaptic firing rate, the pulse width modulation (i.e., firing modulation) of neuronal cells has a significant impact on synaptic weight change. The observed FDP with different EPSC gains can be explained by the competition between the synaptic excitation and memory consolidation processes, which are related to the long-term learning rules. In this work, the long-term synaptic excitation learning process (Abraham et al., 1987) results in the nonvolatile synaptic weight change by an application of well-defined spike train with an amplitude of 4 V in BFO-based artificial synapse. Memory consolidation (Squire et al., 2015) describes the retention of synaptic weight change across time. Synaptic consolidation is a sophisticated process in biological memory trace, where the synaptic weight change overtime could be increased (Bi and Poo, 1998), decreased (Markram et al., 1997), or even unchanged after the initial weight induction (Froemke and Dan, 2002). In BFO-based artificial synapse, it has been demonstrated that the STDP learning functions can be preserved across time up to at least 5 h without collapse, and the slight weight degradation can be observed especially shortly after the application of the initial weight (Du et al., 2015). In Figure 6A, the main spike trains are inducing the variation of synaptic excitation process by sharing the same pulse interval *t*_int_ = 100 ms with different potentiating pulse widths *t*_p_ = 10, 40, 60, 80, and 100 ms. Each positive spike in the spike train will push oxygen vacancies in BFO thin film toward the BE interface and form the un-rectifying region. This will induce the high synaptic weight. The positive spike with larger pulse width *t*_p_ = 100 ms causes significantly higher EPSC gain as more oxygen vacancies are forced close to the BE area. Thus, the depression effect can be observed along the increasing frequency range (blue curve in Figure 6G). In comparison to that, in Figure 6B, the main spike trains are keeping the same pulse width *t*_p_ = 100 ms with various pulse intervals *t*_int_ = 10, 40, 60, 80, and 100 ms. The various pulse intervals *t*_int_ highlight the current degradation effect in the retention test and induce variation of the consolidation process in BFO-based artificial synaptic device. The reduction of conductivity is observed due to the relaxation process, i.e., diffusion of oxygen vacancies away from the BE interface, which increases the barrier height at the bottom interface. Such relaxation process of the oxygen vacancies in the BFO thin film starts along the interval time *t*_int_ and ends until the next stimuli arrive. The longer pulse interval *t*_int_ = 100 ms causes the lowest EPSC gain in Figure 6G (red curve), i.e., depressed synaptic weight, and the potentiation effect can be recorded along the examined increasing frequency range. As a conclusion, for FDP, both depression and potentiation effect for the first time can be obtained within the same frequency range by modulating the ratio between *t*_p_ and *t*_int_ in artificial synapse.

### Application in Unconventional Computing

Inspired by the biological understanding of synaptic and neuronal behaviors in the human brain, the memristive device offers a promising basis for the development of efficient artificial building blocks for brain-inspired unconventional computational paradigms due to their intrinsic properties, such as nonvolatility (no standby power requirement), reconfigurability (simplification of analog circuitry), and strong nonlinear dynamical behavior (full emulation of biological synaptic behavior), which are helpful for solving the latency and power limitations that we face with standard approaches in the modern computer system.

The synaptic and neuronal activities in the biological brain are incredibly slow. The neurons can only fire a few hundred spikes per second, and such electrical stimuli propagate on axons with a velocity of 1–2 m/s. The analog BFO memristive spike-driven circuitry is several orders of magnitude faster (Du et al., 2015) and, thus, could emulate the bio-inspired system much faster than biological realizations. The digital memristive devices (Siemon et al., 2015; Xu et al., 2015), which are normally based on the filamentary switching mechanism, are in general switching faster than analog memristive devices. As experimentally observed in niobium oxide-based memristive devices (Pickett and Williams, 2012), the SET process can be fulfilled at subnanosecond times with 30 nm radius of filamental conduction path. Such switching velocity is expected to be depressed down to 10s of picosecond switching time with 10 nm radius. However, it is also notable that most of the digital memristive devices suffer from various variabilities and defects, which deteriorate the accuracy of the computing system. The demonstrated analog BFO artificial synapses possess ultrastable switching behavior (Figure 1). During the learning process in biological systems, they can change their synaptic strength upon proper electrical stimuli and demonstrate multiple stable resistive states within their dynamic range, which enhances the overall reliability of the brain-inspired computing system. Therefore, by exploiting the memristor-oriented brain-inspired learning approach, it will yield revolutionary results in comparison with conventional CMOS electronics or even outperform the latency performance of the biological human brain.

Besides that, the energy cost of synaptic activities is also critical for evaluating the performance of a brain-inspired computing system. The reported standard CMOS-based artificial synapse usually operates at ∼ nanojoule level per synaptic event (Painkras et al., 2013). The memristive artificial synapses can easily reach several picojoules per synaptic event (Yu et al., 2011; Jackson et al., 2013), or even several hundreds of femtojoules (Xiong et al., 2011; Pickett and Williams, 2012), which is close to the biological brain. To construct the brain-inspired computing system with 10^15^ synapses, the power consumption of synaptic operations by exploiting memristive devices can be significantly decreased by orders of magnitudes in comparison with standard CMOS technology. Furthermore, the single pairing STDP in Figure 3 reveals highly configurable weight change under only signal pairing pre- and postspikes with a wide range of time *t*_p_ and amplitude *V*_p_ according to long-term learning rules (realization of more efficient learning rules). It will be helpful to accomplish the online classification in an accelerated manner and further interact with real-time learning system with reduced energy consumption.

Finally, the implementation of noisy input on BFO memristive device reveals the response of artificial synapse to neuronal noise. The influence of neuronal noise could be beneficial or hinder the functionality of HW-NN. Thus, such study would be also important for applying the memristive artificial synapse as connector in brain-inspired computing systems. The demonstrated robustness of STDP (Figure 7A) from BFO memristive artificial synapses up to a noise level of 30% highlights the use of BFO memristive artificial synapses in NN being part of neuroimplants. This motivates the development of NN in analog hardware with large energy efficiency and speed and robustness against noise propagating through the NN.

## Conclusion

The demand for low-cost brain-inspired unconventional computing has dramatically increased with the rise of big data and the Internet of things. In this work, the BFO-based memristive device is proposed for emulating the functionalities of biological synapse, which is the key component for information transmission in biological human brain. So far, the noisy input data, e.g., from neuroimplants, have not been processed in brain-inspired computing. By the application of the quasi-static stimulation protocol, the STDP learning functions under single pairing spike sequences without and with extrinsic neuronal noisy input are comparatively and experimentally investigated. The highly configurable weight change with a considerable wide range of learning windows in STDP is revealed toward the realization of efficient learning rules. The perfect functioning STDP demonstrated up to a noise level of 30% indicates that analog BFO memristive artificial synapses in NN can be quite resilient toward extrinsic neuronal noise. Moreover, the generalized FDP is analyzed in dependence of the pulse interval time within the same frequency range, and it demonstrated for the first time that synaptic potentiation and depression can be realized within the same firing frequency range. As a conclusion, we have experimentally proven that various synaptic plastic behaviors of synaptic connectivity required in brain-inspired computing can be realized from a single BFO-based memristive device and we also showed their potential to provide superior outcomes in comparison with conventional CMOS electronics. Furthermore, the presented comprehensive experimental study allows a straightforward design of unconventional computing systems by exploiting the dynamical synaptic behaviors in BFO memristive devices and paves the way to a low-cost scalable brain-inspired cognitive computing paradigm.

## Data Availability Statement

The original contributions presented in the study are included in the article/supplementary material, further inquiries can be directed to the corresponding author/s.

## Author Contributions

ND and HS conceived the original idea and developed the methodology. ND analyzed and interpreted the results, and drafted and revised the manuscript. XZ measured and analyzed the experimental results. ZC setup the experimental system and testing programs. MD contributed to guidance and developing different concepts of unconventional computing. BC contributed to fruitful discussions on building memristor models with memristor variability for hardware neural networks. IS and DB prepared the testing memristive chips. All authors contributed to the article and approved the submitted version.

## Conflict of Interest

The authors declare that the research was conducted in the absence of any commercial or financial relationships that could be construed as a potential conflict of interest.
